# Two Programmed Cell Death Systems in *Escherichia coli*: An Apoptotic-Like Death Is Inhibited by the *mazEF*-Mediated Death Pathway

**DOI:** 10.1371/journal.pbio.1001281

**Published:** 2012-03-06

**Authors:** Ariel Erental, Idith Sharon, Hanna Engelberg-Kulka

**Affiliations:** Department of Microbiology and Molecular Genetics, Institute for Medical Research Israel-Canada, The Hebrew University-Hadassah Medical School, Jerusalem, Israel; Baylor College of Medicine, United States of America

## Abstract

In eukaryotes, the classical form of programmed cell death (PCD) is apoptosis, which has as its specific characteristics DNA fragmentation and membrane depolarization. In *Escherichia coli* a different PCD system has been reported. It is mediated by the toxin–antitoxin system module *mazEF*. The *E. coli mazEF* module is one of the most thoroughly studied toxin–antitoxin systems. *mazF* encodes a stable toxin, MazF, and *mazE* encodes a labile antitoxin, MazE, which prevents the lethal effect of MazF. *mazEF*-mediated cell death is a population phenomenon requiring the quorum-sensing pentapeptide NNWNN designated Extracellular Death Factor (EDF). *mazEF* is triggered by several stressful conditions, including severe damage to the DNA. Here, using confocal microscopy and FACS analysis, we show that under conditions of severe DNA damage, the triggered *mazEF*-mediated cell death pathway leads to the inhibition of a second cell death pathway. The latter is an apoptotic-like death (ALD); ALD is mediated by *recA* and *lexA*. The *mazEF*-mediated pathway reduces *recA* mRNA levels. Based on these results, we offer a molecular model for the maintenance of an altruistic characteristic in cell populations. In our model, the ALD pathway is inhibited by the altruistic EDF-*mazEF*-mediated death pathway.

## Abstract 2

A newly discovered apoptotic-like death is inhibited by the previously described mazEF-mediated death pathway, revealing two programmed cell death systems in *Escherichia coli*.

## Author Summary

The enteric bacterium *Escherichia coli*, like most other bacteria, carries on its chromosome a pair of genes, *mazE* and *mazF* (*mazEF*): *mazF* specifies a toxin, and *mazE* specifies an antitoxin. Previously, we have shown that *E. coli mazEF* is responsible for bacterial programmed cell death in response to stressors such as DNA damage. Here, we report that extensive DNA damage can induce a second mode of cell death, which we call apoptotic-like death (ALD). ALD is like apoptosis—a mode of cell death that has previously been recorded only in eukaryotes. During ALD, the cell membrane is depolarized, and the DNA is fragmented and can be detected using the classical TUNEL assay. The MazEF death pathway, however, shows neither of those features, yet also kills the cell. We show that ALD is mediated by two proteins, RecA and LexA, which are noteworthy because LexA is an inhibitor of the SOS response (which is a global response to DNA damage in which the cell cycle is arrested and DNA repair is induced). This defines ALD as a form of SOS response. Furthermore, MazEF and its downstream components cause reduction of recA mRNA levels, which could explain how the MazEF pathway inhibits the ALD pathway. We conclude that the *E. coli* ALD pathway is a back-up system for the traditional *mazEF* cell death pathway. Should one of the components of the *mazEF* pathway be inactivated, bacterial cell death would occur through ALD. These findings also have implications for the mechanisms of “altruistic” cell death among bacterial populations.

## Introduction

Programmed cell death (PCD), defined as an active process that results in cell suicide, is an essential mechanism in multicellular organisms. Generally, PCD is required for the elimination of superfluous or potentially harmful cells (for reviews see [1],[2]). In eukaryotes, the classical form of PCD is called apoptosis [3], a term that originally defined the morphological changes that characterize this form of cell death. Today, the phrase PCD is used to refer to any form of cell death mediated by an intracellular program, no matter what triggers it and whether or not it displays all of the characteristics of apoptosis (for reviews see [1],[2],[4]–[6]).

In bacteria, the best studied PCD systems are mediated through genetic modules. These modules, called “addiction modules” or toxin–antitoxin (TA) systems, consist of a pair of genes that encode two components: a stable toxin and an unstable antitoxin that interferes with the lethal action of the toxin. Initially, such genetic systems for bacterial PCD were found mainly in *Escherichia coli* on low-copy-number plasmids, where they are responsible for what is called the post-segregational killing effect. When a bacterium loses such a plasmid (or other extra-chromosomal element), the cell dies because the unstable antitoxin is degraded faster than is the more stable toxin (reviewed by [7]–[12]). The cells can be thought of as “addicted” to the short-lived product, since its de novo synthesis is essential for their survival. Thus, addiction modules maintain the stability in the host of the extra-chromosomal elements on which they are borne.

TA systems, some of which are homologous to these extra-chromosomal addiction modules, have been found on the chromosomes of many bacteria [13]–[15]. In *E. coli* several well-established TA systems have been described [16]–[22]. Each of these modules consists of a pair of genes, usually co-transcribed as operons, in which the downstream gene encodes a stable toxin and the upstream gene encodes a labile antitoxin. One of the most commonly found and extensively investigated of these modules is the chromosomal TA system *mazEF*, which has been studied mainly in *E. coli* [14]. *E. coli mazF* specifies for the stable toxin MazF, and *mazE* specifies for the labile antitoxin MazE, degraded by ClpPA protease [16]. *E. coli* MazF is a sequence-specific endoribonuclease [23], and *E. coli mazEF* is triggered by stressful conditions, such as DNA damage [24],[25]. We have recently shown that MazF cleaves at ACA sites at or closely upstream of the AUG start codon of some specific mRNAs and thereby generates leaderless mRNAs. Moreover, MazF also targets 16S rRNA within 30S ribosomal subunits at the decoding center, thereby removing the anti-Shine-Dalgarno (aSD) sequence that is required for translation initiation on canonical mRNAs. Thus, under stressful conditions when MazF is triggered, an alternative translation machinery is generated. It consists of a subpopulation of ribosomes that selectively translate leaderless mRNAs [26]. This stress-induced translation machinery is responsible for the selective synthesis of specific proteins caused by MazF induction [27]. In addition, *mazEF*-mediated cell death in *E. coli* is a population phenomenon requiring the presence of the linear pentapeptide NNWNN, which is a quorum-sensing factor called Extracellular Death Factor (EDF) [28],[29]. We have recently shown that EDF induces the endoribonucleolytic activity of *E. coli* MazF [30]. Here we asked: are the characteristics of the EDF-*mazEF*-mediated cell death pathway similar to some of those of eukaryotic apoptosis [1],[2],[4]–[6]?

## Results

### The *mazEF*-Mediated Cell Death Pathway Prevents the Effect of DNA Damage on Membrane Depolarization in *E. coli*

**Figure 1:**
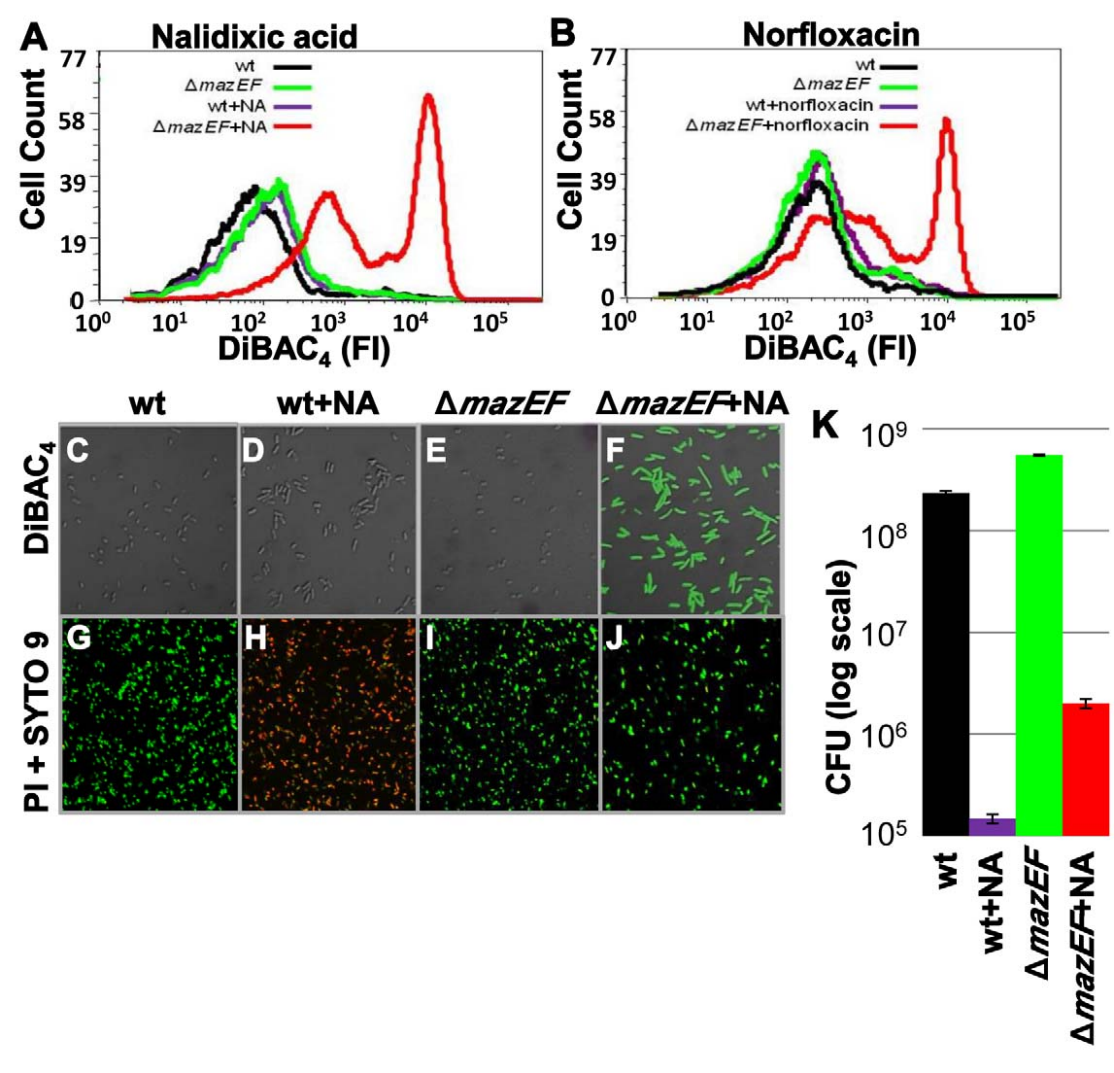
The effect of DNA damage on membrane depolarization and viability. The effect on membrane depolarization (detected by DiBAC_4_) (A–F), cell viability (detected by Live/Dead Kit) (G–J), and CFU (K). *E. coli* strains MC4100*relA* ^+^ (WT) or MC4100*relA* ^+^Δ*mazEF* were grown to OD_600_ 0.6. Each culture was divided into two aliquots, and then the appropriate antibiotics were added to one of the samples: (A) NA (100 µg/ml) or (B) norfloxacin (1.5 µg/ml). Cells were incubated for 4 h and then stained with DiBAC_4_. (A and B) The intensity of the fluorescence (FI) of DiBAC_4_ in the cells was determined by FACS. The fraction of cells in which membrane depolarization occurred was determined as being above 10^3^ DiBAC_4_. (C–F) The fluorescence intensity of DiBAC_4_ in the cells was also determined by confocal microscopy. (G–J) Confocal microscopy of the cells treated with NA as in (C–F), but stained with the Live-Dead Kit. (K) The effect of NA on CFU. All data are representative of three independent experiments.

In eukaryotes, one of the early events in mitochondrial apoptosis is membrane depolarization, involved both in activating the caspase cascade and in opening permeability transition pores [31],[32]. Membrane depolarization can be monitored by using the membrane potential (Δϕ)–sensitive dye bis-1,3-dibutylbarbituric acid trimethine oxonol (DiBAC_4_), which was also previously shown to be a sensitive, robust bacterial viability probe that can be used with flow cytometry [33],[34]. Here, in order to follow membrane depolarization, we used DiBAC_4_ staining together with flow cytometry to enable us to detect fluorescence in individual dying cells. As previously, we triggered *mazEF*-mediated cell death by causing DNA damage by adding nalidixic acid (NA) [35] or norfloxacin [36]. Neither NA nor norfloxacin led to membrane depolarization in wild-type (WT) *E. coli* MC4100*relA* ^+^ cells (Figure 1A and 1B). However, to our surprise, in *E. coli* MC4100*relA* ^+^Δ*mazEF* cells, we observed membrane depolarization after 4 h of treatment with NA (100 µg/ml) (Figure 1A) or norfloxacin (1.5 µg/ml) (Figure 1B), but not at all in untreated cells (Figure 1A and 1B). Only DNA damage led to membrane depolarization and only in the Δ*mazEF* derivative strain; treating WT or Δ*mazEF* derivative cells with an inhibitor of transcription (rifampicin) or an inhibitor of translation (spectinomycin) did not affect membrane depolarization (Figure S1). Thus, when triggered by DNA damage, *mazEF* prevented membrane depolarization. Note that this effect was obtained only when applying NA at a concentration higher than 50 µg/ml (Figure S2B). The prevention of membrane depolarization by *mazEF* was further supported by direct microscopic assessment using DiBAC_4_ to stain cultures of MC4100*relA* ^+^ or MC4100*relA* ^+^Δ*mazEF* cells treated with NA or norfloxacin. Microscopic analysis of these cells stained with DiBAC_4_ (Figures 1C–1F and S3A–S3H) revealed that only the Δ*mazEF* strain treated with NA (Figures 1F and S3H) or with norfloxacin (Figure S3D) appeared green. These results are in contrast to those obtained by direct microscopic assessment using the Live/Dead Kit [37],[38] (Figures 1G–1J and S3K–S3N). After treatment with NA for 4 h, most of the population of WT MC4100*relA* ^+^ cells appeared as dead cells (red) (Figures 1H and S3L), while that of the Δ*mazEF* derivative appeared as live (green) (Figures 1J and S3N). These results suggest that there may be at least two death pathways in *E. coli*, both triggered by DNA damage. One pathway is *mazEF*-mediated, resulting in damage to the cell membrane that permitted the penetration of propidium iodide (PI) (Live/Dead Kit [37],[38]), staining the cells red (Figures 1H and S3L). The second pathway led to membrane depolarization, so the cells were stained green by DiBAC_4_ (Figures 1F, S3D, and S3H). This second pathway was inhibited when the *mazEF*-mediated pathway was triggered by DNA damage (Figures 1D, S3B, and S3F). Survival assays supported the existence of this second death pathway. Treating the cells with 100 µg/ml NA resulted in reducing the viability of the WT MC4100*relA* ^+^ cells to about 0.06% survivors, and reducing the viability of the Δ*mazEF* derivative cells to about 0.36% survivors (Figure 1K).

**Figure 2 pbio-1001281-g002:**
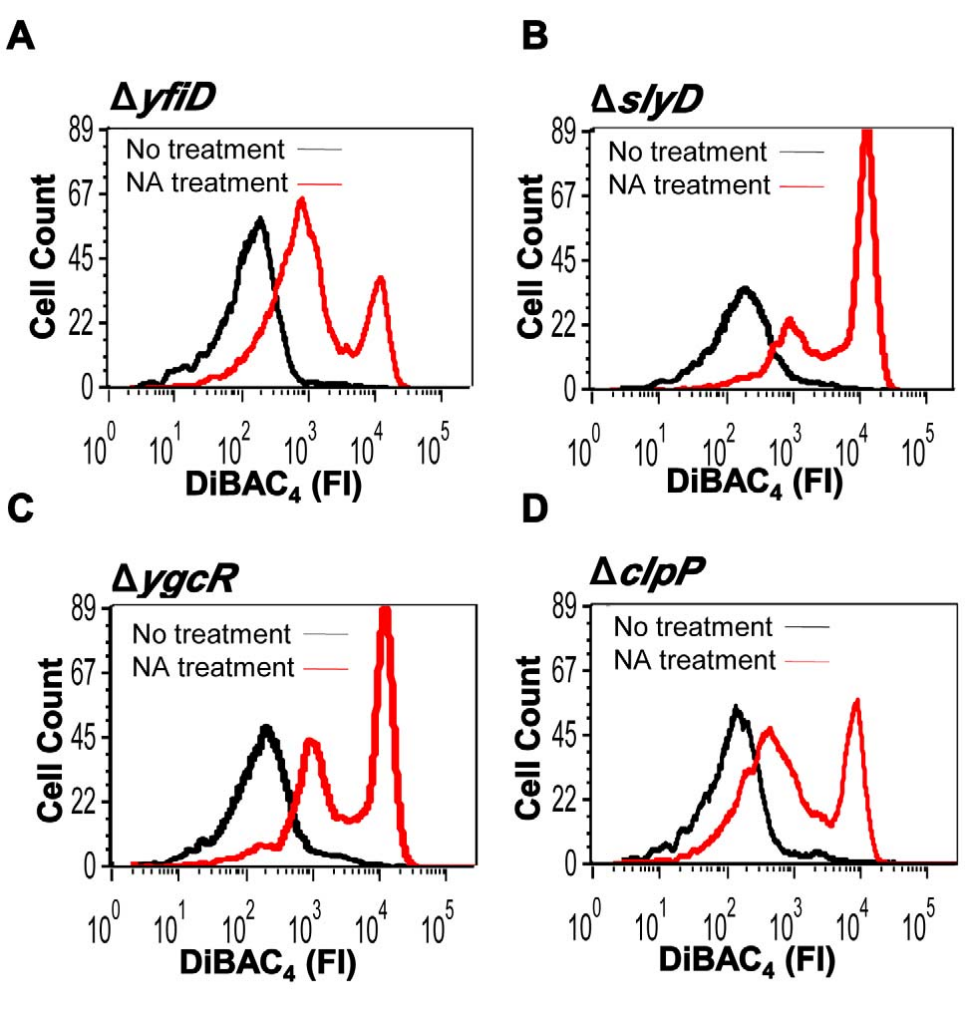
Deleting genes acting downstream from *mazF* leads to membrane depolarization. Cultures of WT *E. coli* MC4100*relA* ^+^ and of derivative strains from which we deleted (A) *yfiD*, (B) *slyD*, (C) *ygcR*, and (D) *clpP* were grown, treated with NA (100 µg/ml), and stained with DiBAC_4_ as described in Figure 1. Samples treated with NA are shown in red, and samples without treatment are shown in black. FI, fluorescence intensity.

Previously, we have reported that when *E. coli* cells are triggered by DNA damage, MazF induces a selective synthesis of proteins specified by the genes *yfiD*, *slyD*, *ygcR*, and *clpP* [27]. As we have more recently shown, these proteins are selectively synthesized by an alternative translation system that is generated by MazF induction under stressful conditions [26]. Since the synthesis of these proteins is generated because of the action of MazF, they are designated by us as the “MazF-downstream system,” and the corresponding genes as the “*mazF*-downstream genes.” Here we asked: would deleting each of these downstream genes permit the operation of the ALD pathway under conditions of DNA damage? To this end, we deleted each of these genes individually from *E. coli* MC4100*relA* ^+^ cells, and treated each deletion strain with NA. Deleting each of the genes *yfiD* (Figure 2A), *slyD* (Figure 2B), *ygcR* (Figure 2C), and *clpP* (Figure 2D) enabled membrane depolarization triggered by NA. The effect of NA on colony-forming units (CFUs) in these strains is presented in Figure S4. In the case of Δ*slyD*, Δ*yfiD*, and Δ*clpP* cells, only 10% of cells survived. Most similar to Δ*mazEF* was Δ*ygcR*, with only about 1% survival. It seems, therefore, that *ygcR* has a more profound effect on cell death than the other studied downstream genes (Figure S4), an issue that is under our investigation. Might *E. coli* TA systems other than *mazEF*, such as *chpBIK*, *relBE*, *dinJ-yafQ* or *yefM-yoeB* [12], also prevent membrane depolarization? Deleting each of those four TA systems did not result in membrane depolarization (Figure S5), suggesting that probably only *mazEF* prevents membrane depolarization.

### The *mazEF*-Mediated Cell Death Pathway Prevents the Effect of DNA Damage on DNA Fragmentation in *E. coli*

**Figure 3:**
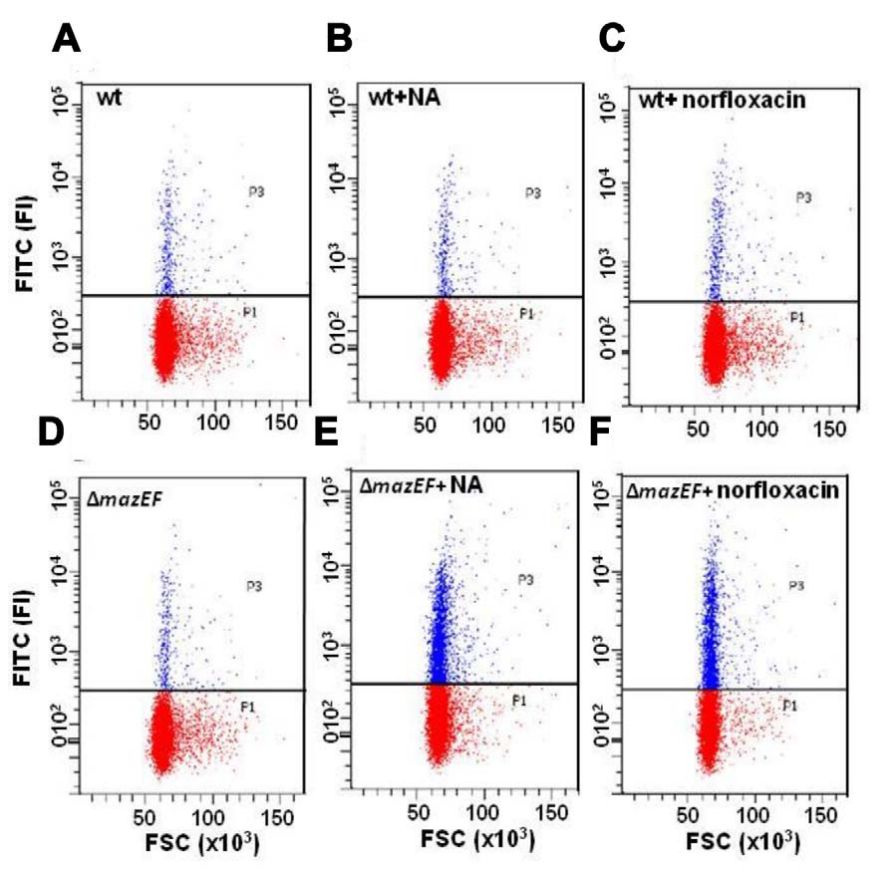
The effect of DNA damage on DNA fragmentation (detected by TUNEL assay). *E. coli* (A–C) MC4100*relA* ^+^ (WT) or (D–F) MC4100*relA* ^+^Δ*mazEF* were grown to OD_600_ 0.6. Each culture was divided into three samples: (A and D) without treatment; (B and E) 100 µg/ml NA was added, and (C and F) 1.5 µg/ml norfloxacin was added. DNA fragmentation was determined using the Apo-Direct Kit. The location of the horizontal threshold line between TUNEL negative cells (highlighted in red) and TUNEL positive cells (highlighted in blue) was determined based on untreated and unstained MC4100*relA* ^+^ cells. All data are representative of three independent experiments. FI, fluorescence intensity; FSC, forward scatter.

Another essential characteristic of apoptosis in eukaryotes is DNA fragmentation, generally detected by the well-known TUNEL assay [39]. Here we asked: when DNA damage triggers *mazEF*, might the action of the *mazEF* system also prevent DNA fragmentation? We followed DNA fragmentation with the TUNEL assay using the Apo-Direct Kit [39], and detected fluorescence in individual dying cells by flow cytometry (Figure 3). In both strains, MC4100*relA* ^+^ and its Δ*mazEF* derivative, a low level of DNA fragmentation was observed, even in untreated cells (Figure 3A and 3D). However, as in the case of membrane depolarization (Figures 1 and S3), the effect of NA and norfloxacin on DNA fragmentation was observed only in the Δ*mazEF* derivative (Figure 3E and 3F) and not in the WT strain (Figure 3B and 3C). The lack of significant DNA fragmentation in the WT *E. coli* MC4100*relA* ^+^ strain treated with either NA (Figure 3B) or norfloxacin (Figure 3C) suggests that the observed DNA fragmentation is not a direct physical manifestation of NA or norfloxacin.

### The “Apoptotic-Like *E. coli* Death Pathway” Is Mediated by *recA* and *lexA*

**Figure 4:**
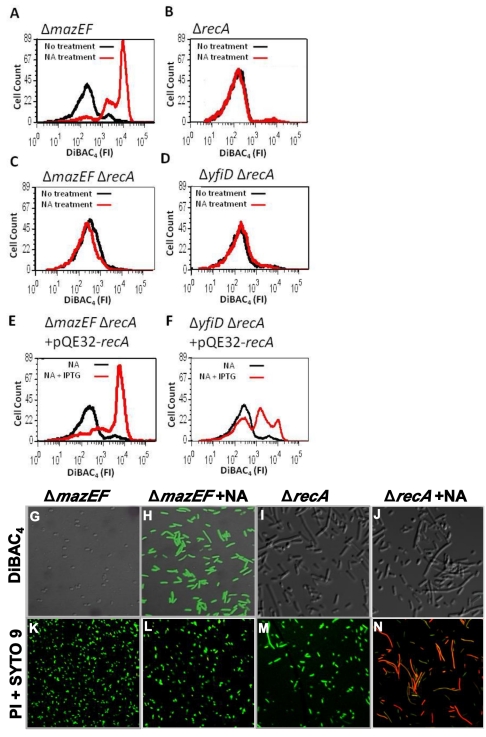
The effect of DNA damage on membrane depolarization is **recA**-dependent. (A–D) *E. coli* (A) MC4100*relA* ^+^Δ*mazEF*, (B) MC4100*relA* ^+^Δ*recA*, (C) MC4100*relA* ^+^Δ*mazEF*Δ*recA*, and (D) MC4100*relA* ^+^Δ*yfiD*Δ*recA* cells were grown, treated with NA (100 µg/ml), and stained with DiBAC_4_ as in Figure 1. Samples treated with NA are shown in red. Control samples without treatment are shown in black. (E and F) Complementation assay with plasmid pQE32-*recA*. *E. coli* strains (E) MC4100*relA* ^+^Δ*mazEF*Δ*recA* and (F) MC4100*relA* ^+^Δ*yfiD*Δ*recA* harboring pQE32-*recA* were grown and treated with NA (100 µg/ml) and in the presence (red) or absence (black) of 1 mM IPTG. The cultures were stained with DiBAC_4_, and the fluorescence intensity (FI) was determined by FACS as described in Figure 1. (G–J) The fluorescence intensity of DiBAC_4_ in cells of MC4100*relA* ^+^Δ*mazEF* and MC4100*relA* ^+^Δ*recA* that were treated with NA was also determined by confocal microscopy. (K–N) Confocal microscopy of the cells treated with NA as in (G–J), but stained with the Live/Dead Kit. All data are representative of three independent experiments.

Since both membrane depolarization and DNA fragmentation were caused in the Δ*mazEF* derivative only by DNA damage, we suspected that ALD is connected to *recA*, a system well known to be involved in the SOS response [40]–[43]. Deleting *recA* from the WT MC4100*relA* ^+^ strain did not permit membrane depolarization, as manifested by DiBAC_4_ staining (Figures 4B and S3J). However, deleting *recA* from MC4100*relA* ^+^Δ*mazEF* prevented membrane depolarization (Figure 4C). In addition, recently, we found that *yfiD* specified for a death protein that acted the farthest downstream from *mazEF* (data not shown). As in the case of Δ*mazEF*, deleting *recA* from the derivative strain Δ*yfiD* also prevented membrane depolarization (Figure 4D). Moreover, when these Δ*recA* strains harbored plasmid pQE32-*recA*, which bears an isopropyl-β-D-thio-galactopyranoside (IPTG)–inducible *recA* gene, and in the presence of IPTG, the membrane became depolarized (Figure 4E and 4F), suggesting that ALD was mediated through *recA*. On the other hand, *recA* is not involved in the *mazEF*-mediated death pathway; deleting *recA* does not prevent the operation of *mazEF*-mediated death. The addition of NA to the MC4100*relA* ^+^Δ*recA* strain still permitted its staining by PI, included in the Live/Dead Kit (Figures 4N and S3P). On the other hand, DiBAC_4_ staining (characteristic for ALD) is prevented in this strain (Figures 4J and S3J). These results also shed light on another aspect of DiBAC_4_ staining. Although Δ*mazEF* cells induced by DNA damage are longer than their WT counterparts (Figures 1D, 1F, S3B, S3D, S3F, and S3H), this is not the reason for the uptake of DiBAC_4_ by these cells. This is demonstrated by the fact that Δ*recA* cells, which appear to be even longer than Δ*mazEF* cells, are not stained by DiBAC_4_ but rather PI (red) (see Figures 4G–4N, S3I–S3J, S3O, and S3P).

We show that activation of the ALD pathway is under the control of the SOS DNA damage response. Under conditions of DNA damage, RecA acts as a co-protease that promotes the autoproteolytic cleavage of LexA, the transcriptional repressor of the SOS response genes, thereby activating the SOS response [40]–[43]. Therefore, we examined whether RecA operates in the ALD pathway in the stage of LexA cleavage. To this aim, we constructed a *lexA3* mutant of *E. coli* strain MC4100*relA* ^+^Δ*mazEF*. *lexA3* encodes a non-cleavable LexA repressor [44]. We found that, as in the case of *E. coli* mutant MC4100*relA* ^+^Δ*mazEF*Δ*recA* (Figure 4C), also the cells of mutant MC4100*relA* ^+^ *lexA*3Δ*mazEF* are not stained by DiBAC_4_ (Figure S6B), indicating that the ALD pathway is induced by inactivation of the repressor LexA by RecA, i.e., an SOS response (Figure S6).

### The Quorum-Sensing Factor EDF Is Involved in *mazEF*-Mediated Inhibition of ALD

**Figure 5:**
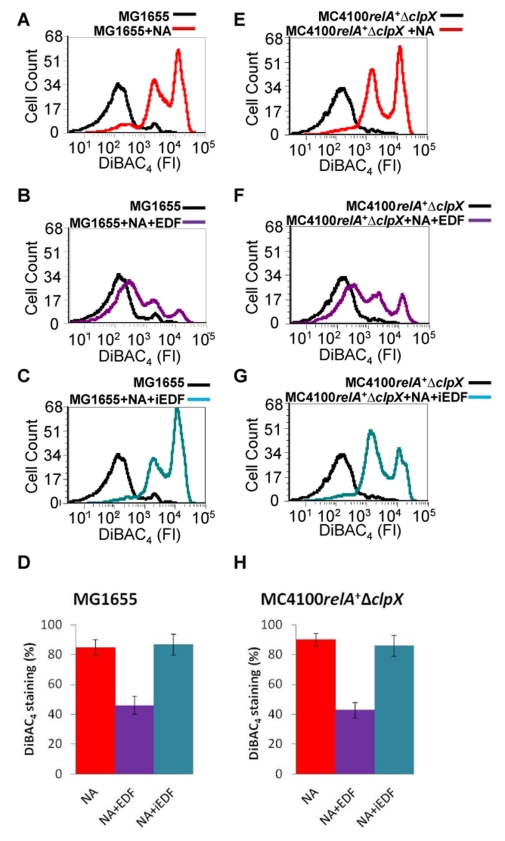
The effect of EDF on membrane depolarization. After the various *E. coli* strains were grown to OD_600_ 0.6, the cells were treated as follows: (A) MG1655+100 µg/ml NA; (B) MG1655+100 µg/ml NA+50 ng/ml EDF; (C) MG1655+100 µg/ml NA+50 ng/ml iEDF (NNGNN); (E) MC4100*relA* ^+^Δ*clpX*+100 µg/ml NA; (F) MC4100*relA* ^+^Δ*clpX*+100 µg/ml NA+200 ng/ml EDF; (G) MC4100*relA* ^+^Δ*clpX*+100 µg/ml NA+200 ng/ml iEDF. The quantitative effect of NA and NA+EDF on the percent of DiBAC_4_-stained cells is shown in (D) (data from [A–C]) and (H) (data from [E–G]). Error bars in (D) and (H) indicate standard deviation. Staining, FACS analysis, and determining the fraction of the DiBAC_4_-stained (membrane depolarized) cells were carried out as described in the legend to Figure 1. All data are representative of three independent experiments. FI, fluorescence intensity.

EDF, which is the quorum-sensing pentapeptide NNWNN, is required for *mazEF*-mediated cell death [28],[29]. Since we have found that *mazEF* as well as its downstream mediated pathway prevent the *recA*-mediated ALD pathway, we asked whether EDF is also involved in this inhibitory effect. To this end we used the *E. coli* MG1655 strain that we previously showed to be defective in EDF production [29]. Using DiBAC_4_ staining and flow cytometry, we observed membrane depolarization in strain MG1655 treated with NA (Figure 5A). However, in the presence of synthetic EDF (50 ng/ml), DiBAC_4_ staining was decreased by about 50% (Figure 5B and 5D), whereas addition of the interfering peptide NNGNN (iEDF) (50 ng/ml) did not affect DiBAC_4_ staining (Figure 5C and 5D). Similar results were obtained using the Δ*clpX* derivative of strain MC4100*relA* ^+^ (Figure 5E–5H) that was previously shown to be defective in EDF production [29]. Thus, the EDF-*mazEF*-mediated cell death pathway is responsible for the inhibition of the ALD pathway.

### The *mazEF*-Mediated Pathway Causes a Reduction in *recA* mRNA Levels

**Figure 6:**
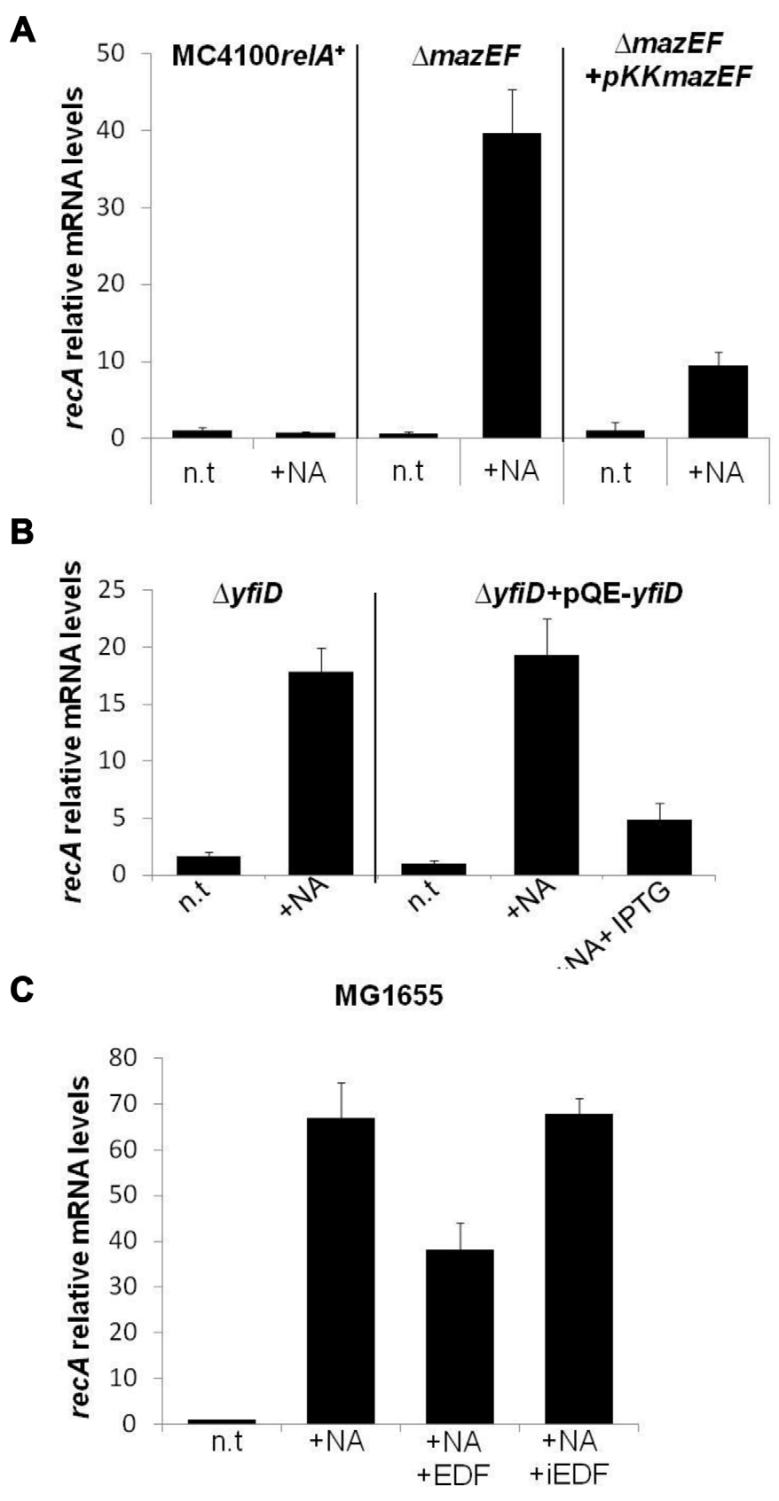
The *mazEF* pathway reduces the levels of *recA* mRNA. (A) *E. coli* cells of strain MC4100*relA* ^+^, its Δ*mazEF* derivative, or Δ*mazEF* carrying plasmid pKK*mazEF* were grown and treated with NA (100 µg/ml) or left without treatment (n.t) as in Figure 1. Then RNA was extracted from the cells, and real-time PCR was performed to quantify *recA* mRNA levels. The indicated values are relative to *recA* RNA levels in untreated MC4100*relA* ^+^ cells. (B) *E. coli* MC4100*relA* ^+^Δ*yfiD* or *E. coli* MC4100*relA* ^+^Δ*yfiD* cells carrying the inducible plasmid pQE-*yfiD* were grown and treated with NA as in (A). To induce the expression of *yfiD*, 1 mM of IPTG was added before the addition of NA. The indicated values are relative to *recA* RNA levels in untreated MC4100*relA* ^+^Δ*yfiD* cells. (C) *E. coli* MG1655 cells were grown and treated with NA (100 µg/ml) or left without treatment as in (A). To test the effect of EDF on this strain, a synthetic EDF (50 ng/ml) or iEDF (50 ng/ml) (NNGNN) was added to the cells before adding the NA. The indicated values are relative to *recA* RNA levels in untreated MG1655 cells. All experiments were performed in triplicate, and a typical experiment out of three is shown. Error bars indicate standard deviation.

Here we asked at what stage is the ALD pathway inhibited by the EDF-*mazEF*-mediated pathway? Since we have found that the ALD pathway is *recA*-dependent (Figure 4), here we examined whether the EDF-*mazEF* pathway affects levels of *recA* mRNA. As shown, the addition of NA to strain MC4100*relA* ^+^ did not increase relative *recA* mRNA levels (Figure 6A). In contrast, these mRNA levels were significantly increased (about 40-fold) by the addition of NA to its Δ*mazEF* derivative (Figure 6A). Moreover, when Δ*mazEF* strains harbored plasmid pKK*mazEF*, which expresses *mazEF* genes constitutively, the relative mRNA levels of *recA* were decreased significantly in the presence of NA (Figure 6A). Since we have previously shown that *yfiD* acts downstream to MazF [26],[27], we here asked whether *yfiD* also reduces the levels of *recA* mRNA. As shown, indeed, the deletion of *yfiD* significantly elevated the level of *recA* mRNA in the presence of NA (Figure 6B). Moreover, when these Δ*yfiD* strains harbored plasmid pQE-*yfiD*, which bears an IPTG-inducible *yfiD* gene, and in the presence of 1 mM IPTG, mRNA levels of *recA* were significantly decreased in the presence of NA (Figure 6B). These results suggest that it is not MazF itself that directly reduces the mRNA levels of *recA*, but rather the product of one of its downstream genes. In addition, we also asked whether EDF is involved in the reduction of *recA* mRNA levels. To this aim, we used the *E. coli* strain MG1655, which is impaired in EDF production [29]. Indeed, the addition of 50 ng/ml synthetic EDF to the MG1655 strain significantly decreased *recA* mRNA levels in the presence of NA, whereas the addition of iEDF (NNGNN) did not affect the levels of this mRNA (Figure 6C).

## Discussion

Here we report that *E. coli* has at least two genetically programmed death pathways. One of them is the EDF-*mazEF*-mediated pathway that was previously described: it is triggered by various stressful conditions [14],[16],[24],[25],[28]. It leads to the death of most of the bacterial population and to the continued survival of a small subpopulation [27]. We have suggested that the EDF-*mazEF*-mediated death of most of the population permits the survival of the small subpopulation that will become a nucleus for the renewed population when conditions are no longer stressful [27]. Thus, we assume that *mazEF*-mediated cell death is actually an altruistic mechanism for bacterial survival under stressful conditions. The small subpopulation of surviving cells will depend on the death of the majority of the population, both for signal molecules and for the nutrients released by the dead cells.

In our work presented here, we found that the EDF-*mazEF*-mediated pathway had yet another important effect. The action of *mazEF* prevented the operation of another bacterial death pathway that would lead to death of the bacterial culture. We designated this second pathway, newly described here, apoptotic-like death (ALD). ALD is *recA*-dependent. Moreover, while EDF-*mazEF* is triggered by many stressful conditions, ALD is triggered only by damage to the DNA (Figures 1–3 and S1). Furthermore, our experiments have revealed that in the ALD pathway RecA operates through its inactivation of the repressor LexA; insertion of a *lexA3* mutation (leading to the formation of a LexA repressor resistant to RecA cleavage) into *E. coli* strain MC4100*relA* ^+^Δ*mazEF* prevents the ALD pathway, as manifested by DiBAC_4_ staining (Figure S6). These results suggest that one or more of the products of the LexA-repressed operons is responsible for bacterial cell death through ALD. A comparison between the SOS and ALD networks, and the conditions required to activate each of them, is under our current investigation. Note that the *recA*-dependent ALD is triggered only under high concentrations of the DNA damaging agent NA (above 50 µg/ml) (Figure S2), indicating that the ALD pathway probably represents a novel form of the SOS response in which the *recA-lexA* system is manifested only under conditions of severe DNA damage. This novel form include DNA fragmentation and membrane depolarization in the Δ*mazEF* derivative (Figures 1 and 3), which are characteristics that were not previously described in the SOS response [40]–[43].

Our experiments have also revealed that while the *mazEF*-mediated pathway inhibits the ALD pathway (Figures 1–3), the latter does not inhibit the *mazEF*-mediated pathway; deleting *recA* still permitted cell death directed by *mazEF* (Figures 4N and S3P). Thus, the *mazEF*-mediated death pathway is *recA*-independent. In addition, interestingly, the *mazEF*-mediated pathway inhibits the *recA*-dependent ALD pathway by reducing the levels of *recA* mRNA (Figure 6). This reduction in mRNA levels may occur either by the inhibition of *recA* transcription or by reducing the stability of *recA* mRNA. The exact mechanism and the component(s) of the *mazEF*-mediated pathway that is responsible for the described effect on *recA* mRNA levels are under our current investigation. Since *recA* transcription is repressed by LexA [40]–[43], we considered the possibility that a component(s) of the *mazEF*-mediated pathway leads to an increase in *lexA* transcription and thereby to the inhibition of *recA* transcription. Such a possibility is already excluded by our experiments (Figure S7; Text S1).

**Figure 7:**
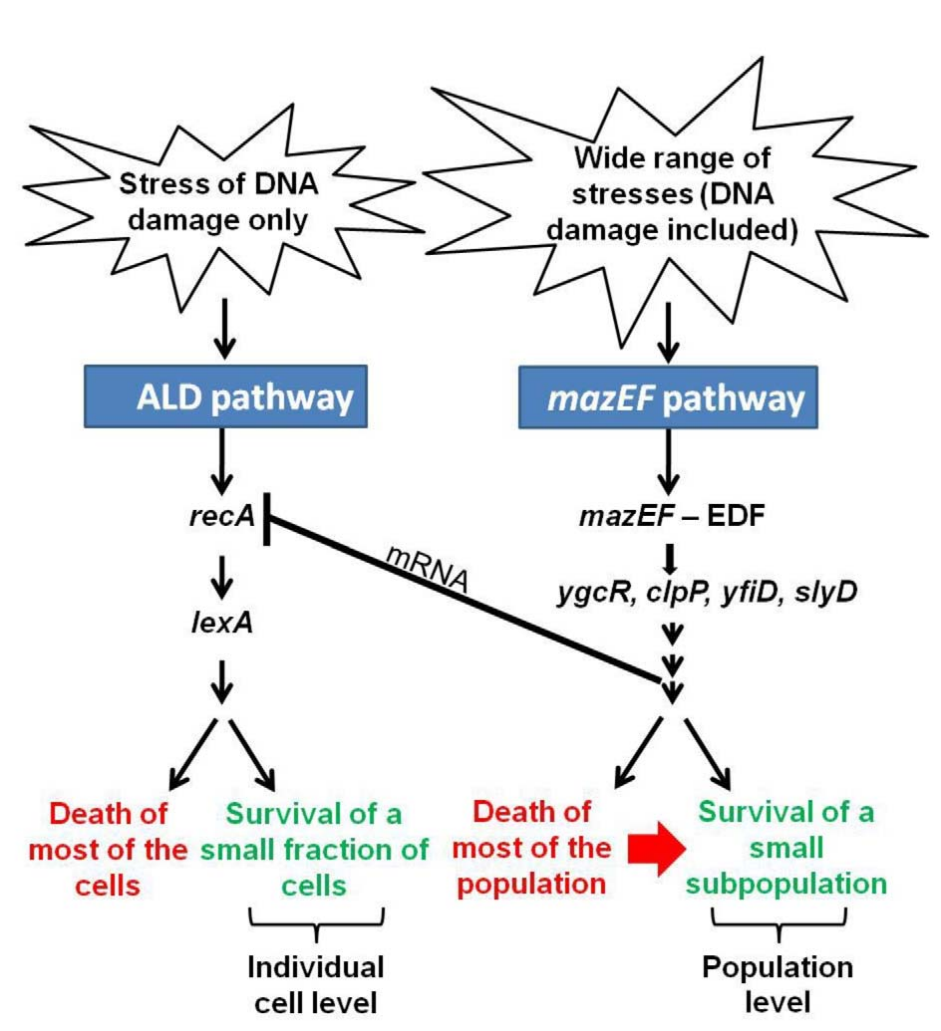
A suggested model for the inhibition of the *recA*- and *lexA*-mediated apoptotic-like pathway by the EDF-*mazEF* pathway. Our model is based on the *mazEF* downstream pathway [27] and the EDF-*mazEF*-mediated cell death pathway [16],[24]–[28] that we have described previously [16],[24]–[28] and on the *recA*- and *lexA*-mediated apoptotic-like pathway on which we have reported here. For additional explanations see also Results and Discussion.

Every living organism must face the challenge of surviving stressful conditions. We assume that in bacteria this function may also be served by genetically programmed cell death pathways. As in case of the EDF-*mazEF* pathway, the ALD death pathway permits cell survival; a small fraction (below 10%) of the cultures still survived after the deletion of either *mazEF* or *recA* (Figure S8). Nevertheless, we suggest that the mechanisms permitting cell survival in the *mazEF* and ALD pathways are operating at two different levels. As described above, the altruistic *mazEF* pathway operates on the level of the bacterial population. Survival of a small subpopulation is due to an altruistic death of most of the population, and is therefore “population dependent” (Figure 7). We suggest that, in contrast, the ALD pathway operates on the level of the individual cell, and therefore it is by definition not altruistic. Survival seems to be due to the repair of individual damaged cells (Figure 7). Moreover, while EDF-*mazEF* is triggered by many kinds of stressful conditions [14],[16],[24],[25],[28], we found here that the ALD pathway is triggered only by DNA damage (Figures 1 and S1). Further studies are needed to examine whether the *mazEF* pathway is indeed a survival solution on the population level while ALD acts only on the level of the individual cell. ALD appears to be similar to eukaryotic apoptosis, because unlike the EDF-*mazEF* pathway, the *recA*-dependent death pathway involves both membrane depolarization and DNA fragmentation (Figures 1 and 3). Further studies will be necessary to clarify whether ALD may share additional characteristics with eukaryotic apoptotic pathways. If so, it may be possible to find some evolutionary connections between eukaryotic apoptotic pathways and the prokaryotic ALD. Recent publications [45]–[48] provide evidence that in eukaryotes, the two PCD pathways, apoptosis and necrosis, are intertwined. More specifically, proteins participating in the apoptotic pathway act together to suppress another type of PCD, necrosis. Clearly, these eukaryotic death pathways are more closely intertwined than was previously thought. Here, to our knowledge for the first time, we show that a similar phenomenon also takes place in prokaryotes, where PCD is not an obvious event, suggesting that the intertwining of PCD systems may be a general biological phenomenon.

We conjecture that ALD may be a back-up system for the EDF-*mazEF*-mediated cell death program. Should one of the components of the EDF-*mazEF*-mediated pathway be inactivated, for example by mutations, leading to the formation of EDF-*mazEF* “cheaters,” bacterial cell death would occur through the ALD pathway. Why might the *mazEF*-mediated death system be preferred over the ALD system? One of the important differences between these two systems is that *mazEF*-mediated death is triggered by a wide range of stressful conditions [14],[16],[24],[25],[28], while as we found here, ALD is triggered only by damage to the DNA (Figures 1 and S1). Thus, cells surviving ALD would subsequently survive only stressful conditions in which the DNA is damaged. On the other hand, cells surviving *mazEF*-mediated death would subsequently survive a wide range of stressful conditions, and thus would have an advantage over the ALD survivors. We suggest that, under the stressful condition of DNA damage, ALD may act as a genetic pressure to maintain the presence of the altruistic EDF-*mazEF*-mediated pathway. In fact, it seems that the EDF-*mazEF* “cheaters” are really cheating themselves: when EDF-*mazEF* cell death is inactivated, the cell population will die because the ALD pathway will no longer be inhibited. Our model (Figure 7) is a sketch of how one efficient cell death pathway can be inhibited by another altruistic death pathway. Ours is the beginning of a model system for “how to maintain an altruistic trait,” a problem that remains a curious and unresolved biological puzzle.

## Materials And Methods

### Bacterial Strains and Plasmids

We used the following *E. coli* strains: MC4100*relA* ^+^ [49] (WT) and its derivatives MC4100*relA* ^+^Δ*mazEF* [49], MC4100*relA* ^+^Δ*clpX* [29], MC4100*relA* ^+^Δ*clpP* [27], MC4100*relA* ^+^Δ*slyD* [27], MC4100*relA* ^+^Δ*yfiD* [27], MC4100*relA* ^+^Δ*ygcR* [27], MC4100*relA* ^+^Δ*relBE* [50], MC4100*relA* ^+^Δ*dinJ-yafQ* [50], MC4100*relA* ^+^Δ*chpBIK* [50], and MC4100*relA* ^+^Δ*yefM-yoeB* [50]. We also used strain MG1655 that we found to be defective in EDF production [29]. In addition, by P1 phage transduction from *E. coli* strain DM2569Δ*recAsrlC300*::Tn10 carrying a *recA* deletion mutation linked to tetracycline resistance [51], we constructed strains MC4100*relA* ^+^Δ*recA*, MC4100*relA* ^+^Δ*mazEF*Δ*recA*, and MC4100*relA* ^+^Δ*yfiD*Δ*recA*. By P1 phage transduction from *E. coli* strain SS2385 *malE*::Tn10 *lexA*3 carrying a *lexA*3 mutation linked to tetracycline resistance [52], we constructed strain MC4100*relA* ^+^ *lexA*3Δ*mazEF* strain. Plasmids pQE32-*recA* and pQE32-*yfiD* are derivatives of pQE32 (Qiagen), which bears *lacI* ^q^ and also bears *recA* or *yfiD* under the control of the *lac* operator and the T5 promoter. Plasmid pKK*mazEF* was described by us previously [28].

### Materials and Media

Bacterial cultures were grown in liquid M9 minimal medium containing 1% glucose and a mixture of 100 µg/ml of each amino acid except tyrosine and cysteine, as previously described [38]. IPTG, NA, norfloxacin, spectinomycin, rifampicin, and ampicillin were obtained from Sigma. Synthetic EDF and synthetic iEDF (NNGNN) (where the central tryptophan of *E. coli* EDF is replaced by glycine) were purchased from GenScript. DiBAC_4_ and the Live/Dead Kit were purchased from Invitrogen. To detect DNA fragmentation, we used the Apo-Direct Kit purchased from Calbiochem.

### Growth Conditions and Viability

For the experiments on membrane depolarization, Live/Dead assays, and cell viability, the cells were grown in 10 ml of M9 minimal medium to OD_600_ 0.5–0.6. Each culture was divided into 500-µl aliquots, to which we added the appropriate antibiotics. These treated aliquots were incubated at 37°C for 4 h, after which they were washed twice with PBS (pH 7.2). To test the effect of the synthetic EDF peptide (NNWNN) or iEDF (NNGNN) on strains MG1655 and MC4100*relA* ^+^Δ*clpX*, we grew the cells as described above; before adding NA to these cultures, we incubated them with synthetic EDF at 37°C for 10 min. For the *recA* or *yfiD* complementation assays, we grew strains MC4100*relA* ^+^Δ*mazEF*Δ*recA* or MC4100*relA* ^+^Δ*yfiD*Δ*recA* or MC4100*relA* ^+^Δ*yfiD*, each hosting the plasmid pQE32-*recA* or pQE32-*yfiD*, in M9 medium as described above, but with the addition of 100 µg/ml ampicillin. To induce the expression of *recA* or *yfiD* immediately before adding NA, we added IPTG to a final concentration of 1 mM. We carried out viability assays on LB plates, as we have described previously [27].

### Membrane Depolarization Assays Using FACS Analysis

Cells were grown, incubated with antibiotics, and washed as described above. Samples were diluted 1∶100 in PBS, and then, to stain the cells, we added 1 µl of DiBAC_4_ (1 mg/ml in ethanol) to 1 ml of the diluted cells. To protect the samples from light, we wrapped the sample tubes in aluminum foil, and incubated them at room temperature for 15 min. We determined the intensity of the fluorescence using a LSRII FACS machine with a 488-nm argon laser for excitation, and a 530±15 nm emission filter, and analyzed the results using FCS Express V3 software.

### DNA Fragmentation Assay Using FACS Analysis

We measured DNA fragmentation using the Apo-Direct Kit, which contains fluorescein isothiocyanate deoxyuridine triphosphate (FITC-dUTP) and the enzyme terminal deoxynucleotidyl transferase. Terminal deoxynucleotidyl transferase adds FITC-dUTP to each 3′-hydroxyl end of fragmented DNA, making it possible to measure the fragmentation of the DNA by the intensity of fluorescence. For our studies on DNA fragmentation, we grew the cells, treated them with antibiotics, and washed them twice with PBS, as described above. Then, for fixation, the cells were re-suspended in 1 ml of 1% paraformaldehyde, incubated for 30 min on ice, centrifuged, washed twice with PBS, re-suspended with 70% cold ethanol, and stored at −20°C overnight. The next day, to prepare the cells for staining, we centrifuged them, gently discarded the ethanol, then re-suspended the cell pellet, and washed the cells twice with 1 ml of the washing buffer from the Apo-Direct Kit. Finally, we re-suspended this washed cell pellet in 50 µl of a staining solution that we composed using the following components from the kit: reaction buffer, FITC-dUTP, and deoxynucleotidyl transferase enzyme dissolved in distilled H_2_O as specified in the kit. We incubated this enzymatic reaction mixture at 37°C for 1.5 h, gently mixing the samples every 15 min. We arrested the reaction by adding 1 ml of the rinse buffer from the kit to each sample, which was then centrifuged and washed once with PBS. After re-suspending the samples in 1 ml of cold PBS, we determined the intensity of the fluorescence by FACS analysis using the LSRII FACS machine and FCS Express V3 software, as described above.

### Confocal Microscopy

To examine the cells using confocal microscopy, we stained them using either DiBAC_4_ or the Live/Dead Kit. The DiBAC_4_ is described above. The Live/Dead kit contains PI and Syto 9. In our experiments, *E. coli* cells were grown, treated with antibiotics, and washed twice with PBS as described above. Then, for DiBAC_4_ staining, 1 µl of DiBAC_4_ (1 mg/ml) in ethanol was added. For the Live/Dead staining, we used 1 µl of a 1∶1 mixture of PI and Syto 9 from the kit after dilution 1∶10. For both DiBAC_4_ and Live/Dead staining, samples were incubated for 15 min at room temperature, and washed twice in PBS. Then, for fixation, 100 µl of 4% formalin was applied for 15 min, and the cells were washed twice with PBS and re-suspended in 20 µl of 50% glycerol. Confocal microscopy was carried out by the use of an Olympus 300 confocal laser scanning microscope with a UPIanSApo 63 oil lens. In order to observe DiBAC_4_ (green) and Syto 9 (green), we used the argon laser with excitation of 488 nm and emission of 515 nm. In order to observe PI (red), we used a HeNe laser with excitation of 543 nm and emission of 570 nm. To compensate for the overlapped wavelength between Syto 9 and PI, we did a sequential scanning.

### Real-Time PCR

For real-time PCR analysis, cells were grown, incubated with antibiotics, and washed as described above. RNA extraction was carried out by using RNAprotect Bacteria reagent (Qiagen) and RNeasy Mini Kit (Qiagen) according to the manufactor's instructions. cDNA was synthesized using the ProtoScript M-MuLV First Strand cDNA Synthesis Kit (New England Biolabs) with random primers (kit component). 16S RNA transcript levels were used as endogenous control for RNA levels in the samples. To amplify *recA* cDNA the following primers were used: forward primer AGATCCTCTACGGCGAAGGT, reverse primer CCTGCTTTCTCGATCAGCTT. To amplify 16S cDNA the following primers were used: forward primer TGTAGCGGTGAAATGCGTAGA, reverse primer CACCTGAGCGTCAGTCTTCGT. To amplify *lexA* cDNA the following primers were used: forward primer GACTTGCTGGCAGTGCATAA, reverse primer TCAGGCGCTTAACGGTAACT. Real-time analysis was then conducted using Fast SYBER Green Master Mix (Applied Biosystems) in a 7500 Fast Real-Time PCR system (Applied Biosystems).

## Supporting Information

Figure S1 **Membrane depolarization was not affected by transcription or translation inhibitors, as detected by DiBAC_4_.**
*E. coli* strains MC4100*relA* ^+^ and MC4100*relA* ^+^Δ*mazEF* were grown as described in Figure 1 and then treated with the translation inhibitor spectinomycin at concentration (A) 50 µg/ml, (B) 100 µg/ml, or (C) 400 µg/ml, or with the transcription inhibitor rifampicin at concentration (D) 50 µg/ml, (E) 100 µg/ml, or (F) 400 µg/ml. Subsequently, the cells were stained with DiBAC_4_ and analyzed by FACS as described in Figure 1.
(TIF)

Figure S2 **The effect of various concentrations of NA on membrane depolarization.**
*E. coli* strains (A) MC4100*relA* ^+^ (WT) and (B) MC4100*relA* ^+^Δ*mazEF* were grown to OD_600_ 0.6. Each culture was divided into aliquots and then the appropriate NA concentration (0, 5, 10, 50, or 100 µg/ml) was added to each of the samples. Cells were incubated and stained with DiBAC_4_, and the intensity of the fluorescence (FI) was determined as in Figure 1.
(TIF)

Figure S3 **The effect of DNA damage on staining by DiBAC_4_ and by the Live/Dead Kit.**
Conofcal microscopy showing the effects of DNA damage on staining by DiBAC_4_ and by the Live/Dead Kit of the WT *E. coli* strain MC4100*relA* ^+^ (A,B,E,F,K, and L), and its derivatives MC4100*relA* ^+^Δ*mazEF* (C,D,G,H,M, and N) and MC4100*relA* ^+^Δ*recA* (I,J,O, and P). DNA damage was caused by either NA (100 µg/ml) or norfloxacin (1.5 µg/ml). This figure shows the same confocal microscopic results as illustrated in Figures 1C–1J and 4G–4N. But here, the confocal images are enlarged, with scale bars included. For additional information see 1C–1J and 4G–4N.
(TIF)

Figure S4 **The effect of deleting genes acting downstream of** ***mazF*** **on the viability of** ***E. coli*** **strain MC4100** ***relA*** **^+^.**
Cultures from *E. coli* strain MC4100*relA* ^+^ and its derivatives Δ*mazEF*, Δ*yfiD*, Δ*slyD*, Δ*ygcR*, or Δ*clpP* were grown and treated with NA (100 µg/ml), and CFU was determined.
(TIF)

Figure S5 **The TA systems** ***dinJ-yafQ*** **,** ***chpBIK*** **,** ***relBE*** **, and** ***yefM-yoeB*** **did not affect membrane depolarization.**
*E. coli* MC4100*relA* ^+^ and derivative strains from which we deleted (A) *mazEF*, (B) *chpBIK*, (C) *relBE*, (D) *dinJ-yafQ*, or (E) *yefM-yoeB* were grown and treated with NA (100 µg/ml) and stained with DiBAC_4_ as described in Figure 1.
(TIF)

Figure S6 **The effect of** ***lexA3*** **mutation on membrane depolarization in strain MC4100** ***relA*** **^+^Δ** ***mazEF*** **.**
*E. coli* strains (A) MC4100*relA* ^+^Δ*mazEF* and (B) MC4100*relA* ^+^ *lexA3*Δ*mazEF* were grown and treated with NA (100 µg/ml) and stained with DiBAC_4_ as in Figure 1. Samples treated with NA are shown in red. Control samples without treatment are shown in black. The cultures were stained with DiBAC_4_, and the fluorescence intensity was determined by FACS analysis as described in Figure 1.
(TIF)

Figure S7 **The reduction in the relative levels of** ***recA*** **mRNA by the** ***mazEF*** **pathway is not due to an increase in** ***lexA*** **transcription.**
*E. coli* MC4100*relA* ^+^ cells were grown and treated with NA (100 µg/ml) or left without treatment (n.t) as in Figure 1. Then RNA was extracted from the cells, and real-time PCR was performed to quantify *lexA* mRNA levels. The indicated values are relative to *lexA* RNA levels in untreated MC4100*relA* ^+^ cells. Experiments were performed in triplicate, and a typical experiment out of three is shown. Error bars indicate standard deviation.
(TIF)

Figure S8 **The effect of deleting** ***recA*** **on the viability of** ***E. coli*** **strain MC4100** ***relA*** **^+^ and its derivatives under conditions of DNA damage.**
*E. coli* MC4100*relA* ^+^ and its derivative strains Δ*mazEF*, Δ*recA*, Δ*mazEF*Δ*recA*, and Δ*yfiD*Δ*recA* were grown and treated with NA (100 µg/ml), and CFU was determined.
(TIF)

Text S1 **Induction of the *mazEF*-mediated pathway does not cause an increase in *lexA* transcription.**
(DOC)

## Abbreviations

ALD: apoptotic-like death
CFU: colony-forming unit
DiBAC_4_: bis-1,3-dibutylbarbituric acid trimethine oxonol
FITC-dUTP: fluorescein isothiocyanate deoxyuridine triphosphate
iEDF: interfering peptide NNGNN
IPTG: isopropyl-β-D-thio-galactopyranoside
NA: nalidixic acid
PCD: programmed cell death
PI: propidium iodide
TA: toxin–antitoxin
WT: wild-type
