# Supplementary figures and images for: Two Programmed Cell Death Systems in Escherichia coli: An Apoptotic-Like Death Is Inhibited by the mazEF-Mediated Death Pathway
Source: PLoS Biol. 2012 Mar 6;10(3):e1001281. doi: 10.1371/journal.pbio.1001281 (PMC3295820; doi:10.1371/journal.pbio.1001281)

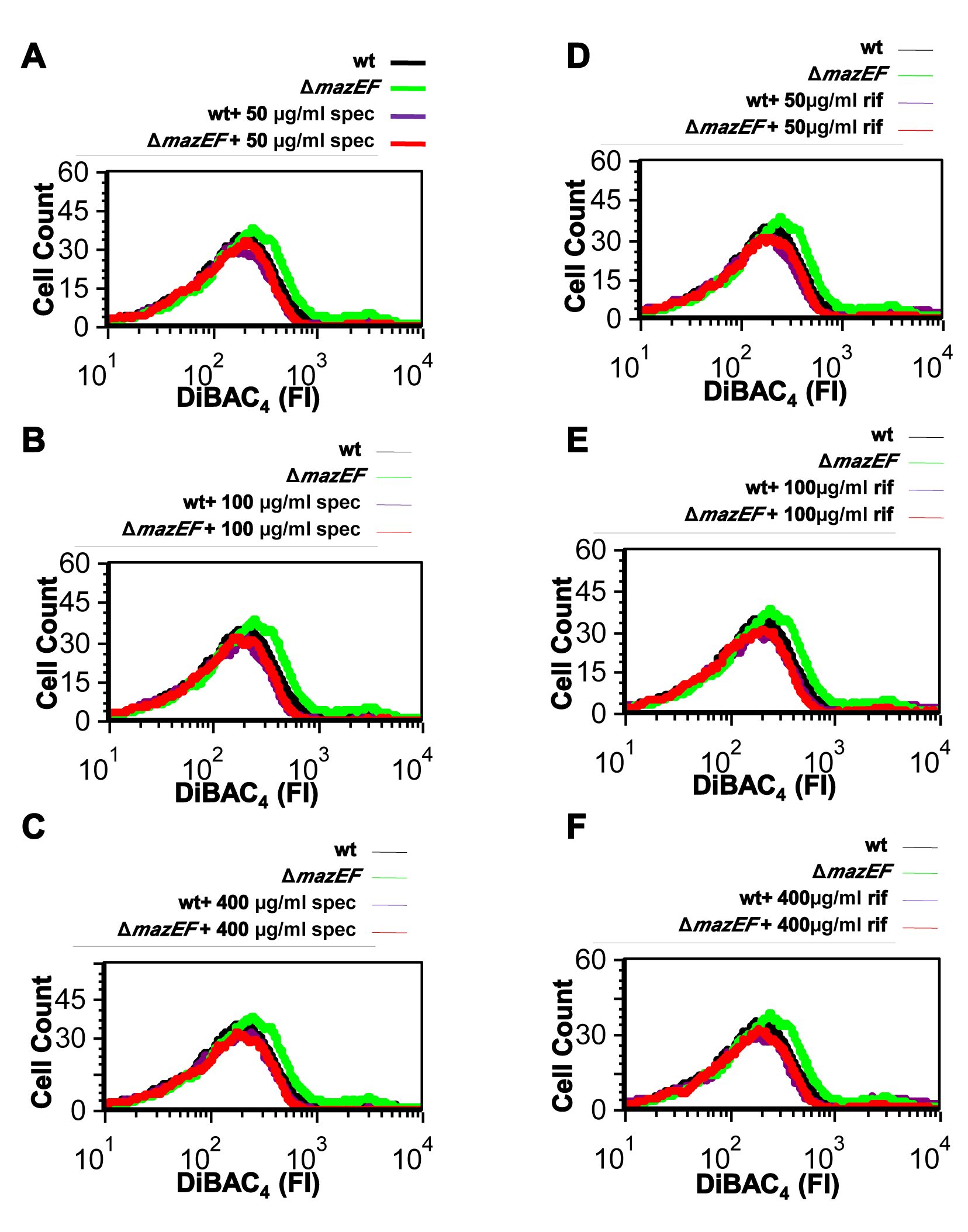

Supplement: Figure S1 — E. coli strains MC4100relA + and MC4100relA +ΔmazEF were grown as described in Figure 1 and then treated with the translation inhibitor spectinomycin at concentration (A) 50 µg/ml, (B) 100 µg/ml, or (C) 400 µg/ml, or with the transcription inhibitor rifampicin at concentration (D) 50 µg/ml, (E) 100 µg/ml, or (F) 400 µg/ml. Subsequently, the cells were stained with DiBAC4 and analyzed by FACS as described in Figure 1. (TIF) [file pbio.1001281.s001.tif]

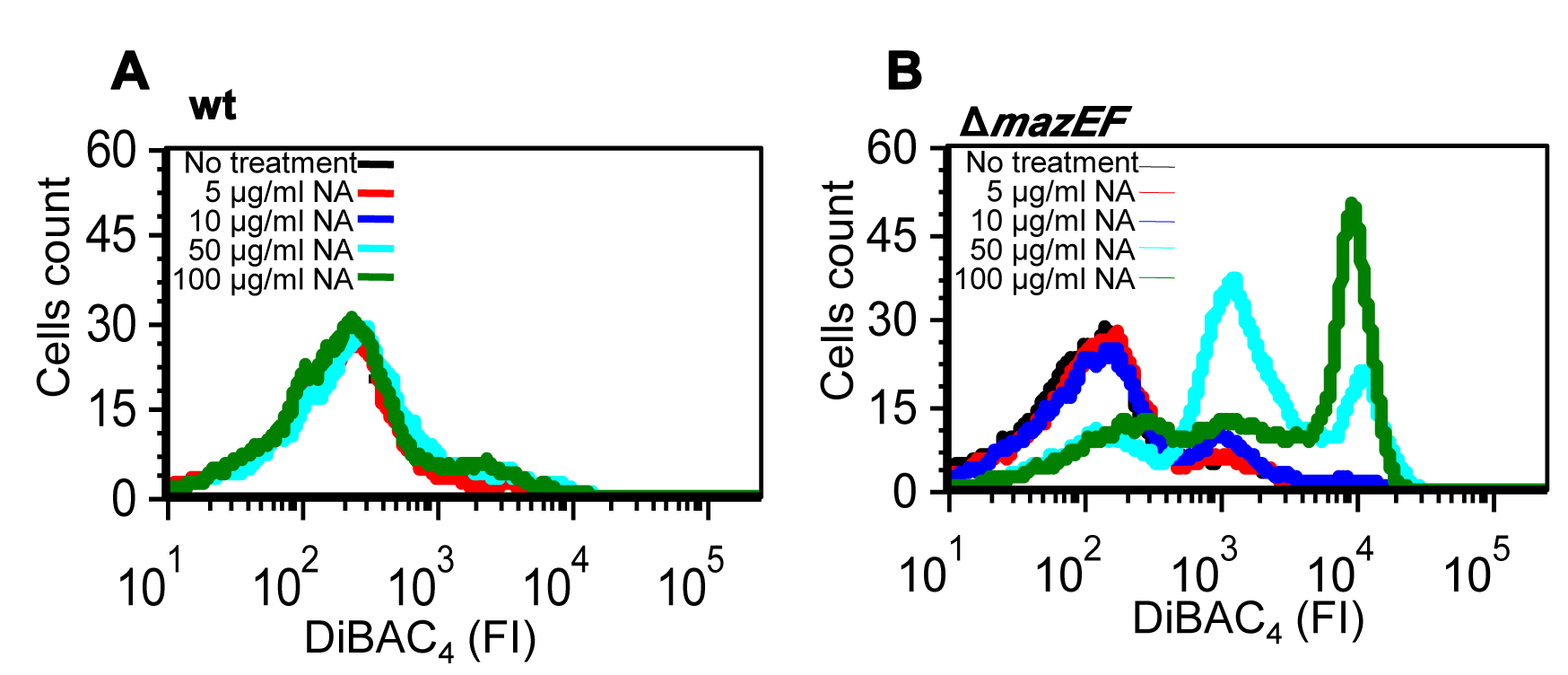

Supplement: Figure S2 — E. coli strains (A) MC4100relA + (WT) and (B) MC4100relA +ΔmazEF were grown to OD600 0.6. Each culture was divided into aliquots and then the appropriate NA concentration (0, 5, 10, 50, or 100 µg/ml) was added to each of the samples. Cells were incubated and stained with DiBAC4, and the intensity of the fluorescence (FI) was determined as in Figure 1. (TIF) [file pbio.1001281.s002.tif]

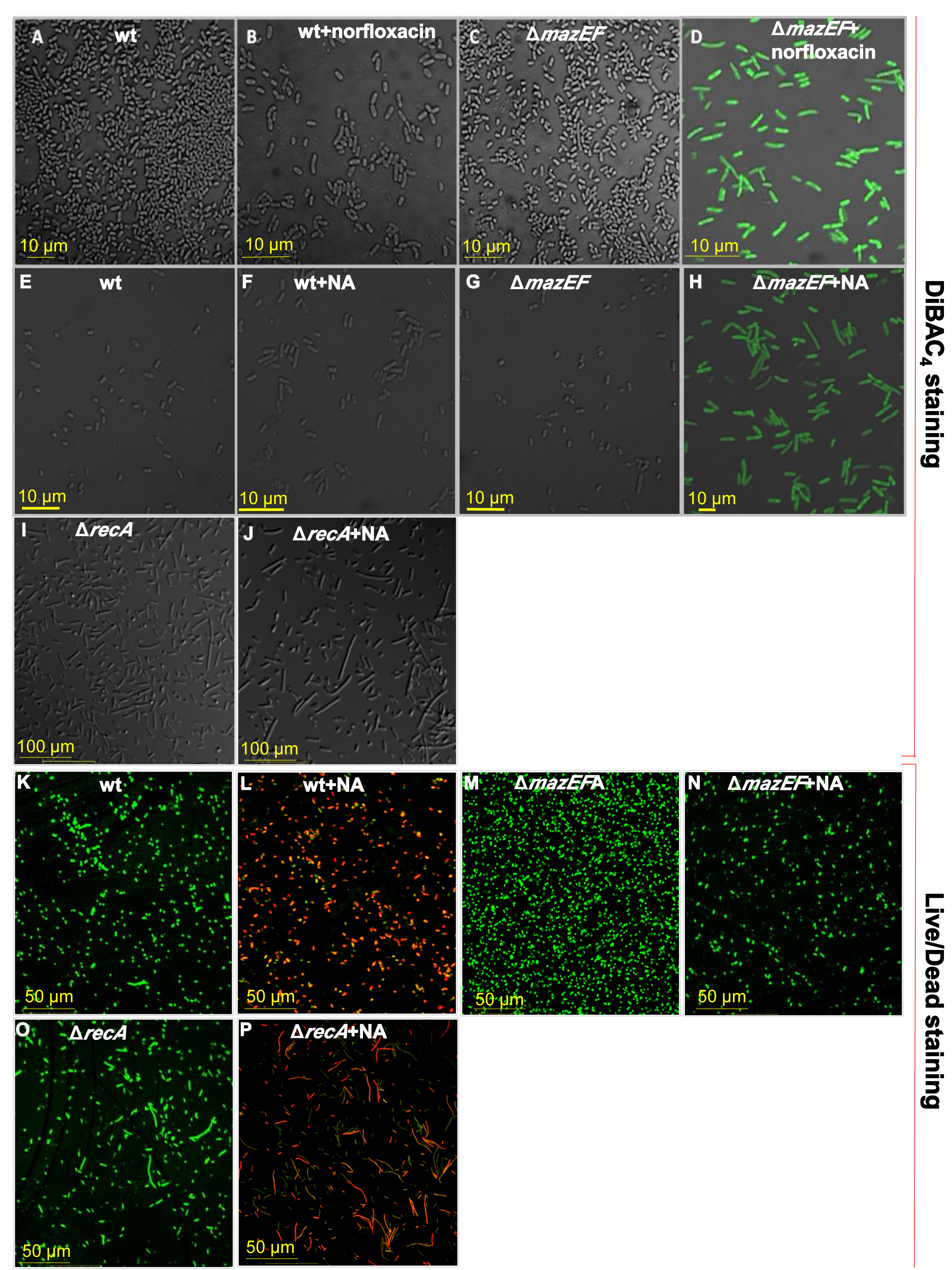

Supplement: Figure S3 — Conofcal microscopy showing the effects of DNA damage on staining by DiBAC4 and by the Live/Dead Kit of the WT E. coli strain MC4100relA + (A,B,E,F,K, and L), and its derivatives MC4100relA +ΔmazEF (C,D,G,H,M, and N) and MC4100relA +ΔrecA (I,J,O, and P). DNA damage was caused by either NA (100 µg/ml) or norfloxacin (1.5 µg/ml). This figure shows the same confocal microscopic results as illustrated in Figures 1C–1J and 4G–4N. But here, the confocal images are enlarged, with scale bars included. For additional information see 1C–1J and 4G–4N. (TIF) [file pbio.1001281.s003.tif]

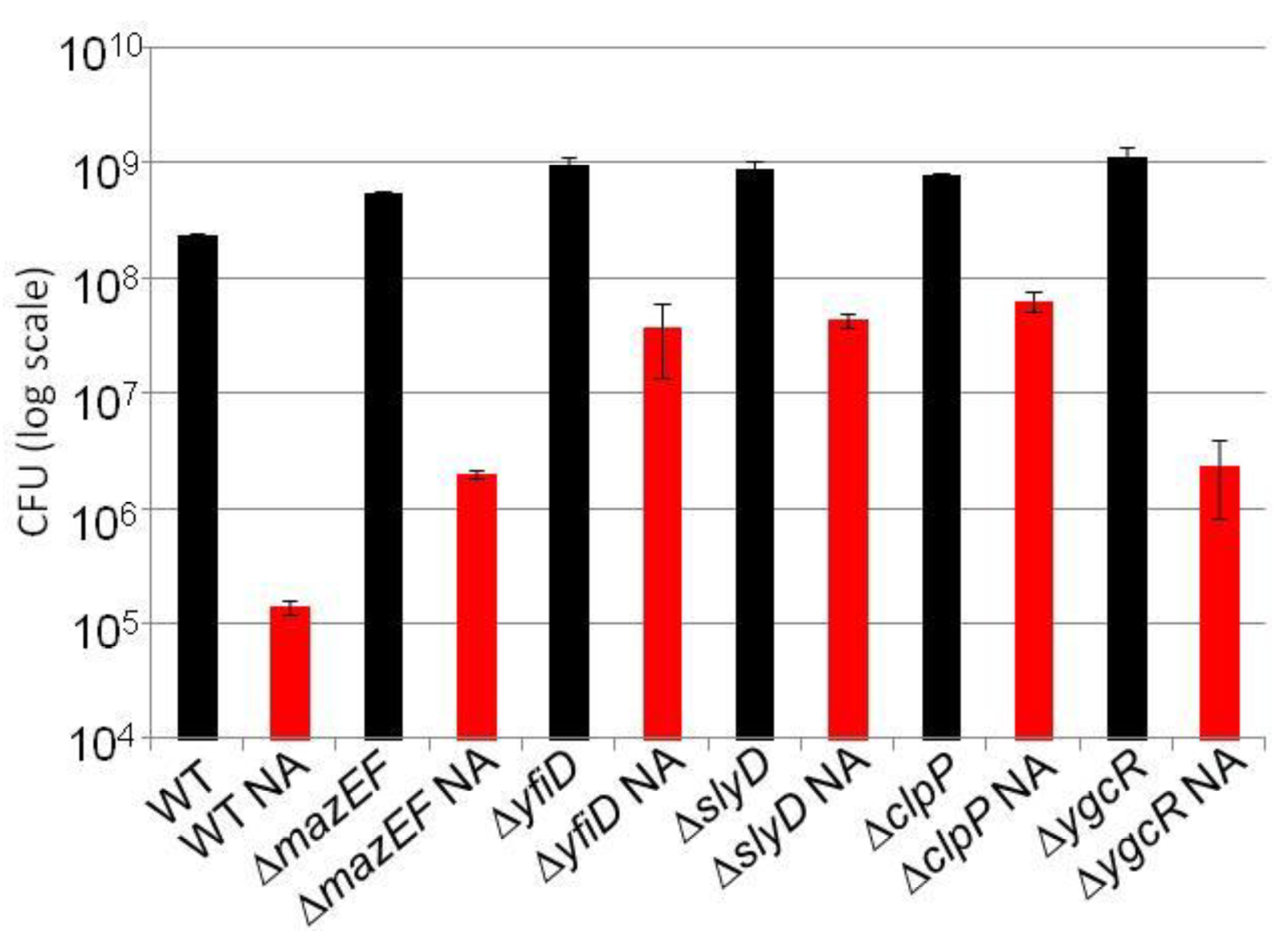

Supplement: Figure S4 — Cultures from E. coli strain MC4100relA + and its derivatives ΔmazEF, ΔyfiD, ΔslyD, ΔygcR, or ΔclpP were grown and treated with NA (100 µg/ml), and CFU was determined. (TIF) [file pbio.1001281.s004.tif]

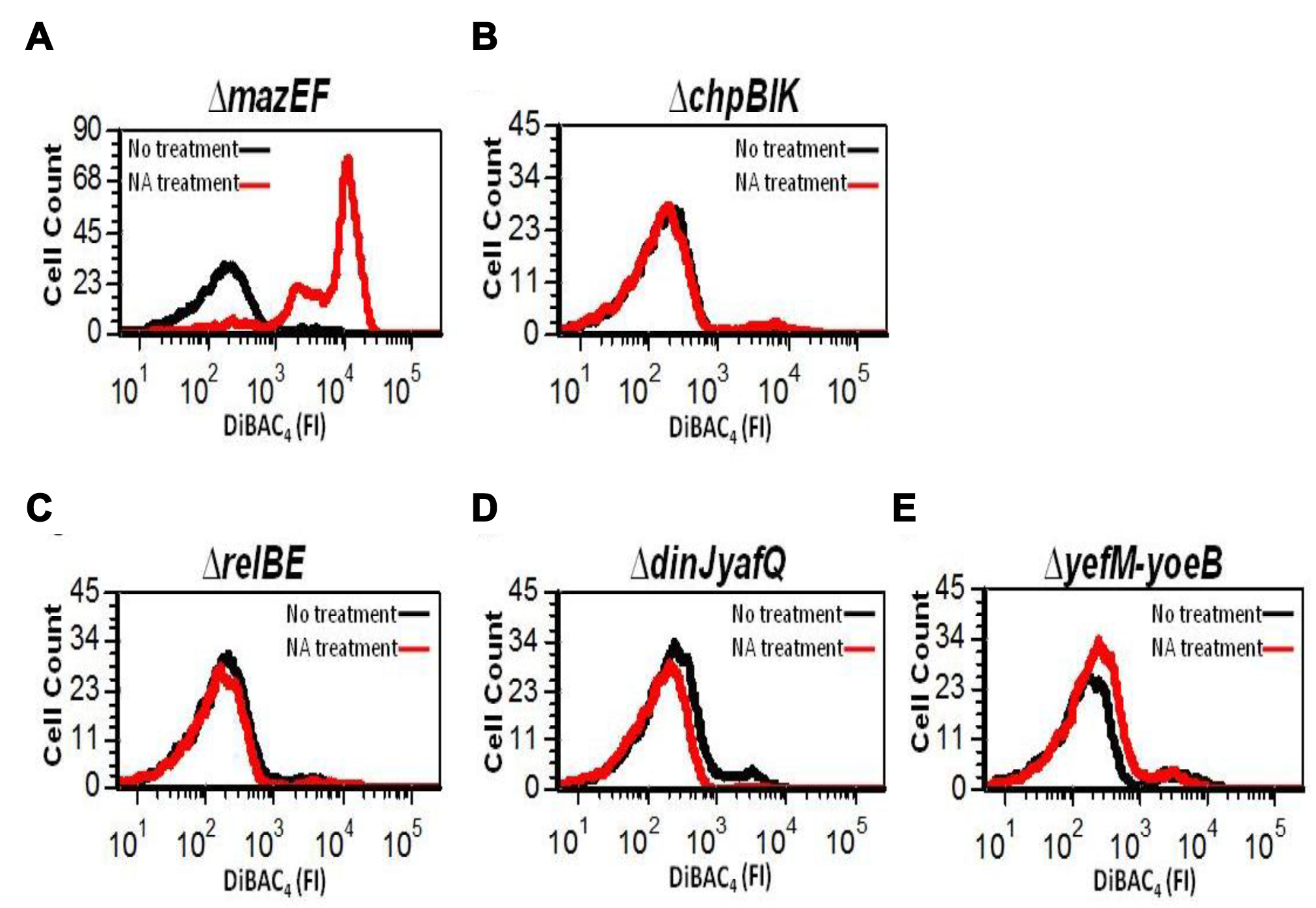

Supplement: Figure S5 — E. coli MC4100relA + and derivative strains from which we deleted (A) mazEF, (B) chpBIK, (C) relBE, (D) dinJ-yafQ, or (E) yefM-yoeB were grown and treated with NA (100 µg/ml) and stained with DiBAC4 as described in Figure 1. (TIF) [file pbio.1001281.s005.tif]

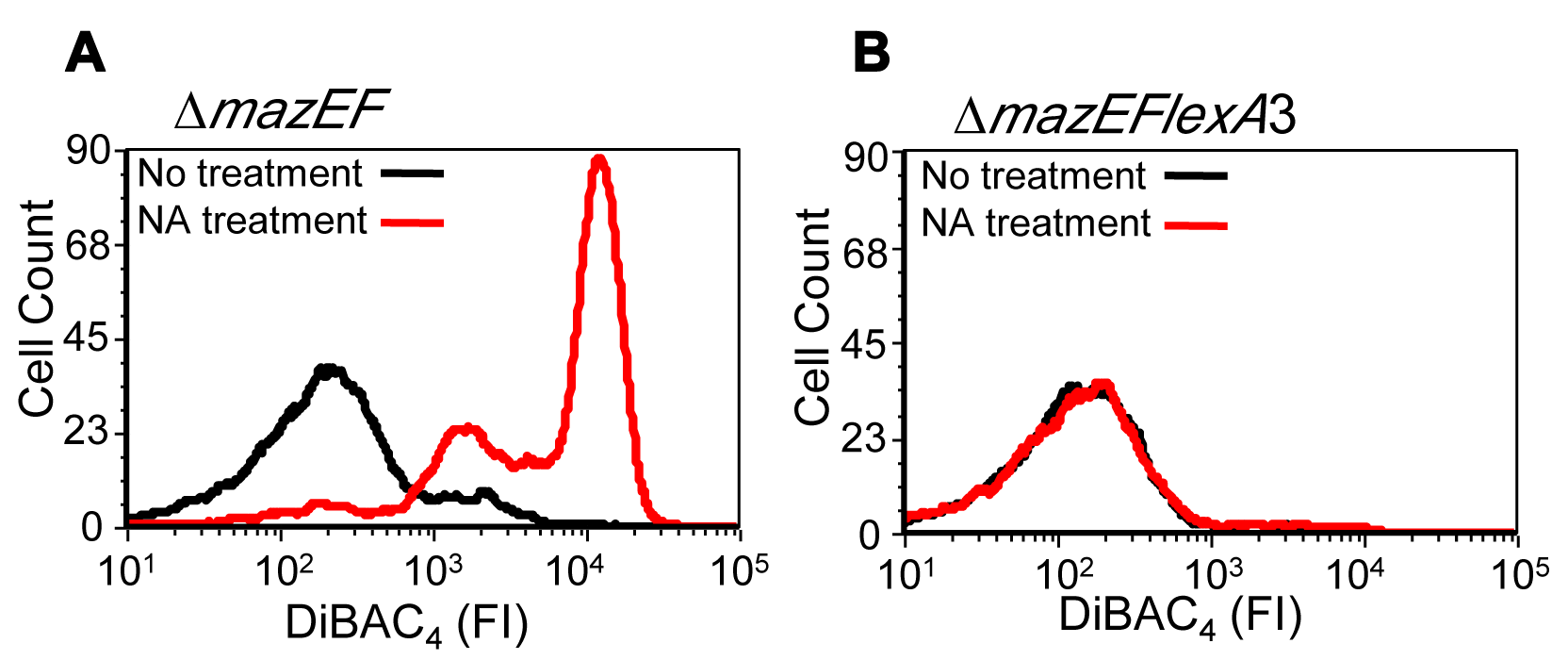

Supplement: Figure S6 — E. coli strains (A) MC4100relA +ΔmazEF and (B) MC4100relA + lexA3ΔmazEF were grown and treated with NA (100 µg/ml) and stained with DiBAC4 as in Figure 1. Samples treated with NA are shown in red. Control samples without treatment are shown in black. The cultures were stained with DiBAC4, and the fluorescence intensity was determined by FACS analysis as described in Figure 1. (TIF) [file pbio.1001281.s006.tif]

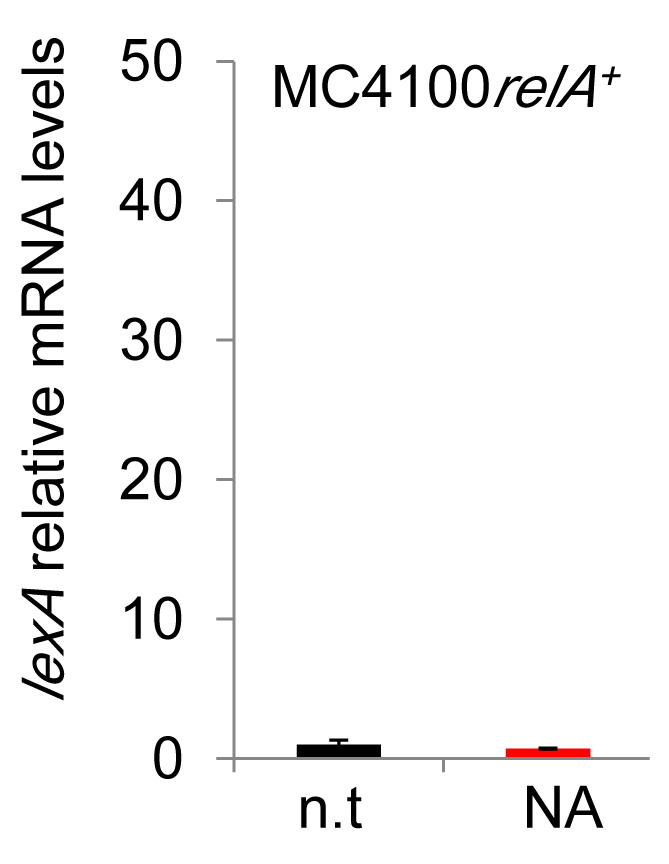

Supplement: Figure S7 — E. coli MC4100relA + cells were grown and treated with NA (100 µg/ml) or left without treatment (n.t) as in Figure 1. Then RNA was extracted from the cells, and real-time PCR was performed to quantify lexA mRNA levels. The indicated values are relative to lexA RNA levels in untreated MC4100relA + cells. Experiments were performed in triplicate, and a typical experiment out of three is shown. Error bars indicate standard deviation. (TIF) [file pbio.1001281.s007.tif]

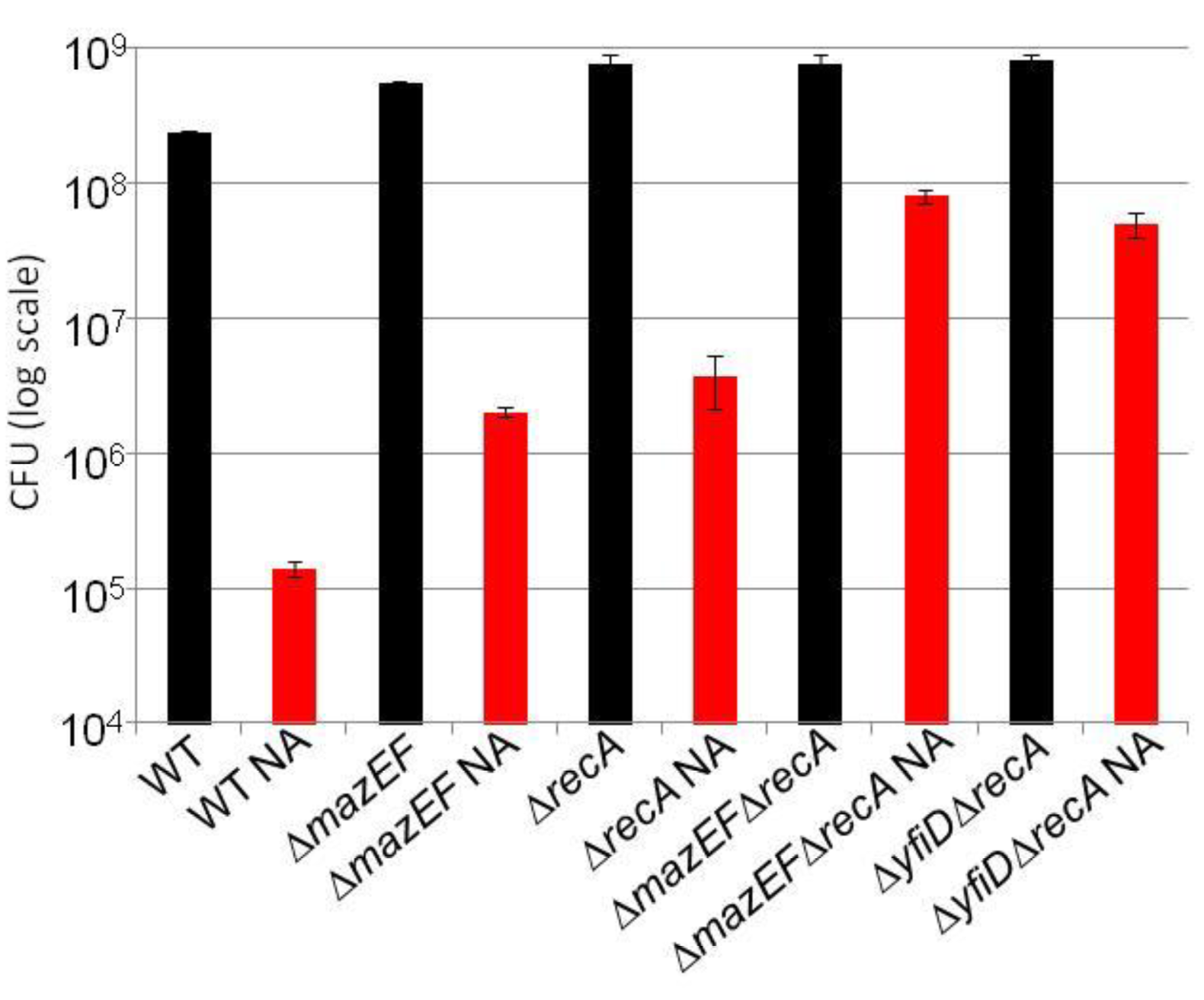

Supplement: Figure S8 — E. coli MC4100relA + and its derivative strains ΔmazEF, ΔrecA, ΔmazEFΔrecA, and ΔyfiDΔrecA were grown and treated with NA (100 µg/ml), and CFU was determined. (TIF) [file pbio.1001281.s008.tif]
