# Supplementary material for: Two Programmed Cell Death Systems in Escherichia coli: An Apoptotic-Like Death Is Inhibited by the mazEF-Mediated Death Pathway
Source: PLoS Biol. 2012 Mar 6;10(3):e1001281. doi: 10.1371/journal.pbio.1001281 (PMC3295820; doi:10.1371/journal.pbio.1001281)
Supplement: Text S1 — (DOC) [file pbio.1001281.s009.doc]

Supporting Information

**Supporting Text**

**Induction of the *mazEF*-mediated pathway does not cause an increase in *lexA* transcription.**

Our experiments have revealed that the *mazEF-*mediated pathway inhibits the *recA*-dependent ALD pathway by the inhibition of *recA* transcription (Figure 6). Since *recA* transcription is repressed by LexA protein [40-43], we asked whether the *mazEF-* mediated pathway causes an increase in *lex*A transcription and thereby to inhibition of *recA* transcription. To this aim, we compared l*exA* transcription in MC4100*relA*^+^ strain with and without NA treatment. No effect on *lexA* transcription was observed (Figure S7).
